# Potential autotrophic carbon-fixer and Fe(II)-oxidizer *Alcanivorax* sp. MM125-6 isolated from Wocan hydrothermal field

**DOI:** 10.3389/fmicb.2022.930601

**Published:** 2022-10-14

**Authors:** Mingcong Wei, Xiang Zeng, Xiqiu Han, Zongze Shao, Qian Xie, Chuanqi Dong, Yejian Wang, Zhongyan Qiu

**Affiliations:** ^1^Ocean College, Zhejiang University, Zhoushan, China; ^2^Key Laboratory of Submarine Geosciences, Second Institute of Oceanography, Ministry of Natural Resources, Hangzhou, China; ^3^Key Laboratory of Marine Biogenetic Resources, Third Institute of Oceanography, Ministry of Natural Resources, Xiamen, China; ^4^School of Oceanography, Shanghai Jiao Tong University, Shanghai, China; ^5^College of Marine Geosciences, Ocean University of China, Qingdao, China

**Keywords:** *Alcanivorax*, autotrophic carbon fixation, Fe(II)-oxidation, *in situ* enrichment, hydrothermal massive sulfide

## Abstract

The genus *Alcanivorax* is common in various marine environments, including in hydrothermal fields. They were previously recognized as obligate hydrocarbonoclastic bacteria, but their potential for autotrophic carbon fixation and Fe(II)-oxidation remains largely elusive. In this study, an *in situ* enrichment experiment was performed using a hydrothermal massive sulfide slab deployed 300 m away from the Wocan hydrothermal vent. Furthermore, the biofilms on the surface of the slab were used as an inoculum, with hydrothermal massive sulfide powder from the same vent as an energy source, to enrich the potential iron oxidizer in the laboratory. Three dominant bacterial families, *Alcanivoraceae*, *Pseudomonadaceae*, and *Rhizobiaceae*, were enriched in the medium with hydrothermal massive sulfides. Subsequently, strain *Alcanivorax* sp. MM125-6 was isolated from the enrichment culture. It belongs to the genus *Alcanivorax* and is closely related to *Alcanivorax profundimaris* ST75FaO-1*^T^* (98.9% sequence similarity) indicated by a phylogenetic analysis based on 16S rRNA gene sequences. Autotrophic growth experiments on strain MM125-6 revealed that the cell concentrations were increased from an initial 7.5 × 10^5^ cells/ml to 3.13 × 10^8^ cells/ml after 10 days, and that the δ^13^C_*VPDB*_ in the cell biomass was also increased from 234.25‰ on day 2 to gradually 345.66 ‰ on day 10. The gradient tube incubation showed that bands of iron oxides and cells formed approximately 1 and 1.5 cm, respectively, below the air-agarose medium interface. In addition, the SEM-EDS data demonstrated that it can also secrete acidic exopolysaccharides and adhere to the surface of sulfide minerals to oxidize Fe(II) with NaHCO_3_ as the sole carbon source, which accelerates hydrothermal massive sulfide dissolution. These results support the conclusion that strain MM125-6 is capable of autotrophic carbon fixation and Fe(II) oxidization chemoautotrophically. This study expands our understanding of the metabolic versatility of the *Alcanivorax* genus as well as their important role(s) in coupling hydrothermal massive sulfide weathering and iron and carbon cycles in hydrothermal fields.

## Introduction

In the deep sea, hydrothermal activities transfer vast fluxes of mass and energy from the solid earth to the oceans, not only feeding massive sulfide deposits but also fostering rich hydrothermal ecosystems (Dick, 2019). As hydrothermal activity wanes or ceases, the massive sulfides would be weathered by the biogeochemical process (Herzig and Hannington, 1995; Hannington et al., 2011; Wang et al., 2017) and become the dominant source of electron donors in the process of chemosynthetic productivity (Sylvan et al., 2012a,b; Van Dover, 2019; Hou et al., 2020). In this process, dominant microorganism communities have gradually changed from “fluid-shaped” to “mineral-shaped” (Hou et al., 2020). Ultimately, class *Gammaproteobateria* becomes the fundamental component of the microbial community inhabiting the hydrothermal massive sulfide (Suzuki et al., 2004; Kato et al., 2017; Van Dover, 2019; Hou et al., 2020).

Members of the *Gammaproteobacteria* class have metabolic and functional versatility in cosmopolitan marine environments, e.g., iron oxidation and carbon fixation (Dickerman et al., 2010; Lesniewski et al., 2012; Van Dover, 2019). Previous results demonstrate that iron oxidizers belonging to *Gammaproteobacteria* are preferentially colonized in metalliferous substrates with high iron concentrations, which suggests a strong correlation between microbes and minerals (Emerson and Moyer, 2002; Kato et al., 2010; Zhang L. et al., 2016; Zhang X. et al., 2016; Li et al., 2017; Van Dover, 2019; Meier et al., 2019; Escudero et al., 2020; Hou et al., 2020). Laboratory and *in situ* incubation experiments reveal that oxidation of seafloor iron-rich substrates releases energy, which is mediated or catalyzed by iron oxidizers and can be harnessed for cellular processes (Eberhard et al., 1995; Edwards et al., 2003b; Emerson and Floyd, 2005; Smith et al., 2011; Callac et al., 2015; Kato et al., 2015; Sudek et al., 2017). Moreover, a metagenomics analysis of microbes colonized on the sulfide mineral surface indicated that autotrophic iron oxidizers in this class can fix inorganic carbon *via* the CBB cycle (Hou et al., 2020). The iron oxidizers in iron-rich habitats affiliated to the class *Gammaproteobacteria* include but are not limited to the genera *Pseudoalteromonas*, *Pseudomonas*, *Halomonas*, and *Marinobacter* (Edwards et al., 2003b; Sudek et al., 2009, 2017; Smith et al., 2011). In the iron-rich environment, these iron oxidizers promote weathering and cycling, affecting the bioavailability and solubility of a variety of elements, such as carbon, sulfur, and iron. Despite the importance and prevalence of these bacteria, studies on iron oxidizers still lag behind other important marine microbes because of limited habitats and difficulties in culturing, isolating, and identifying isolates from iron rich substrates (Emerson et al., 2010). Therefore, more pure culture isolates are required to isolate from iron-rich substrates in deep-sea hydrothermal fields and explore their metabolic potentials (e.g., autotrophy and iron oxidation) in the laboratory.

The genus *Alcanivorax* belonging to the class *Gammaproteobacteria* was proposed by Yakimov et al. (1998) and was gradually recovered and isolated from deep-sea hydrothermal fields (Vetriani et al., 2005; Bertrand et al., 2013; Ramasamy et al., 2020; Dong et al., 2021; Sinha et al., 2021), such as sulfide minerals and hydrothermal vent plume. As far as we know, there are 16 valid types of species in this genus,^1^ 15 of which are isolated from deep sea sediments and seawater (Dong et al., 2021). They have previously been recognized as obligate hydrocarbon decomposers that can utilize alkanes as carbon sources that facilitate the removal of alkanes from marine environments (Yakimov et al., 1998, 2019; Dong et al., 2021; Sinha et al., 2021). Recently, the strain 3B.1 was isolated from a long-term *in situ* enrichment basalt slice at the oceanic crust on Juan de Fuca Ridge and was first classified as an iron oxidizer in the genus *Alcanivorax* (Smith et al., 2011). Because of paucity of enough attention, it is currently the only strain in the genus *Alcanivorax* spp. reported to have an iron oxidation function. Two strains (YD13-A and YDC2-H) belonging to the genus *Alcanivorax* were isolated from hydrothermal sediments that contain massive basalt and black chimney debris using NaHCO_3_ or CO_2_ as a carbon source (Liu et al., 2009). However, they paid more attention to the features in response to heavy metals rather than the function of autotrophic carbon fixation of the *Alcanivorax* species in deep-sea hydrothermal fields. Therefore, two important questions that remain to be addressed are whether the genus *Alcanivorax* can autotrophically fix carbon and whether there exist other strains that can oxidize Fe(II) to support cell growth.

In the present study, a hydrothermal massive sulfide slab (consisting mainly of pyrite) was incubated for 18 months at a distance of approximately 300 m away from the hydrothermal vent on the Carlsberg Ridge (6°21′N, 60°31′E, water depth 2,975 m). A modified MMJHS medium (Takai et al., 2003; Nunoura et al., 2007), with metal sulfide powder as an energy source and NaHCO_3_ as a carbon source, was used to enrich potential chemoautotrophic or chemoheterotrophic iron oxidizers in colonized biofilms from the sample surface. Therefore, the objectives of this study were (i) to assure whether the genus *Alcanivorax* can be enriched and kept steady in the primary and secondary laboratory enrichment cultures, and (ii) to assess the carbon fixation and Fe(II) oxidation potential of strain MM125-6 by physiologic characterization and genome annotation. To our knowledge, this is the first report on autotrophic carbon fixation and Fe(II) oxidization in the genus *Alcanivorax*.

## Materials and methods

### Deep-sea *in situ* incubation

The hydrothermal massive sulfide (Supplementary Figure 1) was collected from the Wocan-1 hydrothermal vent (6°22′N, 60°31′E; water depth, 3,010 m) on the Carlsberg Ridge in the Northwest India Ocean during the DY38th cruise (March to April 2017) with deep manned submersible Jiaolong (CSSC, China) mounted on research vessel *Xiangyanghong* (No. 9). The hydrothermal massive sulfide was cut into slabs with a diamond saw, and its flat sides were glued to glass slides with Super Glue (Supplementary Figure 2). The slab (mainly composed of pyrite) was mounted on a sediment trap (40 m above the seafloor) for *in situ* incubation 300 m away from the Wocan-1 hydrothermal vent on the Carlsberg Ridge (6°21′N, 60°31′E). The sample was deployed on 13 July 2018 (DY49th cruise) and retrieved on 4 November 2019 (DY57th cruise). After retrieval, the slab sample was gently rinsed with sterile seawater and then transferred into a 50-ml falcon tube. The slab was immersed in sterile seawater and slightly shaken and let to stand for 5 min. Then, the slab and the mixture were retrieved and reserved, respectively. The aforesaid mixture was filled with a 10% glycerol TE stock (20 ml 100 × TE buffer, 60 ml deionized water, and 100 ml molecular-grade glycerol; passed the glycerol TE stock through a 2-mm Acrodisc) in sterile seawater onboard and stored at −20^°^C as the enrichment source material until laboratory enrichment. The slab was stored at 4^°^C for cell stain and microscopy observation.

### Enrichment and isolation

In the laboratory, the modified MMJHS medium was used to enrich the iron-or sulfur-oxidizer bacteria that could potentially participate in weathering the hydrothermal massive sulfide in the deep-sea hydrothermal field (Takai et al., 2003). The composition of the MMJHS medium was: NaCl (30 g/L), NH_4_Cl (0.25 g/L), KCl (0.33 g/L), CaCl_2_⋅2H_2_O (0.14 g/L), MgCl_2_⋅6H_2_O (4.18 g/L), K_2_HPO_4_ (0.14 g/L), NaHCO_3_ (1 g/L), Na_2_S_2_O_3_⋅5H_2_O (10 mM), Wolfe’s vitamins (1 ml/L), and trace element solutions (10 ml/L) under a headspace gas mixture with 76% H_2_/20% CO_2_/4% O_2_ (0.25 MPa) (Takai et al., 2003, 2006). For the modified MMJHS medium, the Na_2_S_2_O_3_⋅5H_2_O in the MMJHS medium was replaced with 2 g sterile hydrothermal massive sulfide powder (Supplementary Figure 1; powder size ≤ 150 μm, sulfur element content > 10%). Briefly, 0.2 ml of the mixture sample was immediately inoculated into a 50-ml serum bottle with the modified MMJHS medium and then incubated at 28^°^C for 1 month according to Xie et al. (2021). JL125_P_1 and JL125_P_2 are set as a parallel experimental group for the primary culture. To exclude the influence of relic nucleic acid molecules in the initial inoculum and obtain a stable bacterial community, the primary cultures need to be inoculated into a new medium. Briefly, a 0.2 ml enrichment culture of JL125_P_2 is inoculated into a fresh medium for subculture according to the above method. JL125_S_1 and JL125_S_2 are set as parallel experimental groups in a subculture. At the end of each incubation experiment, a 0.2 ml enriched sample was respectively taken for staining as well as fluorescent microscopy imaging (refer to chapter “Iron oxidation ability assay”). The residue samples used for DNA extraction were immediately frozen at 80^°^C.

To isolate the potential autotrophic bacteria, 0.2 ml of the aforementioned subculture was inoculated into an MMJHS medium and incubated at 28^°^C for 10 days. The pure culture of strain MM125-6 was isolated with the dilution-to-extinction technique (Takai and Horikoshi, 2000; Takai et al., 2003, 2004). The purity of the culture was confirmed by 16S rRNA gene sequencing. Finally, strain MM125-6 was inoculated into a modified MMJHS medium for 3 months to test whether it could grow in an oligotrophic environment using hydrothermal sulfide as an energy source. During and after the experiment, a 0.2 ml sample mixed with powder was taken and stained with SYTO9 to examine cells that were bound to sulfide minerals. The reacted powder sample was lyophilized to analyze the surface morphology of minerals by SEM-EDS (refer to section “Fluorescence microscopy and scanning electron microscopy”).

### 16S rRNA gene clone library construction and sequence analysis

Partial DNA extraction from frozen culture samples was performed with the PowerSoil DNA Isolation Kit (Zymo Research DNA Clean and Concentrator*™*-5; Carlsbad, CA, United States) according to the manufacturer’s instructions. PCR amplification of DNA sequences as well as high-throughput sequencing was accomplished by Shanghai Majorbio Biopharm Technology Co., Ltd (Shanghai, China). PCR was performed using the bacteria-universal primer pair 338F/806R targeting the V3–V4 region of the 16S rRNA gene of bacteria (Caporaso et al., 2011). The amplification conditions and process were performed as described by Zeng and An (2021). The PCR cycle times were set to 35 using the system 9700 (ABI, Foster City, CA, United States), and its conditions were 95^°^C for 30 s, 55^°^C for 30 s, and 72^°^C for 45 s. Amplicons were extracted from 2% agarose gels and purified using the AxyPrep DNA Gel Extraction Kit (Axygen Biosciences, Axygen Biosciences, Union City, CA, United States) following the manufacturer’s recommendations and quantified using QuantiFluor-ST (Promega, Madison, United States). Clone libraries were prepared with the TruSeq™ DNA Sample Prep Kit (Illumina, Madison, WI, United States) according to the manufacturer’s instructions. The nucleotide sequences of randomly selected clones were determined after the PCR products were cloned. The pair-end (PE) reads were merged into the same sequence according to the overlapped relationship between each other. Then, the reads and the effect of mergence were filtered and quality-controlled to obtain the effective sequences. Effective sequences in the samples were distinguished and corrected with the sequences of barcode at pair-end and primer. Nucleotide sequences were aligned using fastp v0.19.4. Finally, high-quality clone libraries were sequenced using Illumina Miseq 2500.

A microbial diversity analysis was performed on the Majorbio cloud platform^2^ (Ren et al., 2022). The Uprase (version 11.0) software was applied to cluster the sequences into operational taxonomic units (OTUs) of > 97% similarity (Edgar, 2013). Based on DOTUR, sequences with not less than 97% similarity were recognized as same phylotypes (Schloss and Handelsman, 2005). The taxonomic position of the representative 16S rRNA gene sequence of each OTU was analyzed with the RDP classifier^3^ using the Silva 16S rRNA database (Release 138) (Pruesse et al., 2007; Quast et al., 2012) and the Greengene database (Release 13.5) (Yokono et al., 2018; Parks et al., 2022) with a 70% confidence threshold. The alpha diversity parameters, including Good’s coverage and Chao 1, ACE, Shannon, and InvSimpson indices, were calculated for each sample with Mothur (Good, 1953; Chao and Bunge, 2002). A composition analysis of the community was performed in R 4.0.3 according to the taxonomic results.

### Phylogeny, genome assemblies, and genomic features of *Alcanivorax* sp. MM125-6

The 16S rRNA gene and genomic sequencing of *Alcanivorax* sp. strain MM125-6 was carried out by Shanghai Majorbio. For this purpose, strain MM125-6 was grown in the MMJHS medium at 28^°^C for 10 days. The high-quality genomic DNA of *Alcanivorax* sp. MM125-6 was extracted and purified using PowerSoil DNA Isolation Kit (Zymo Research DNA Clean and Concentrator*™*-5; Carlsbad, CA, United States) according to the manufacturer’s instructions. The 16S rRNA gene was amplified by PCR with a universal bacterial primer set, 24F and 1492R (Lane et al., 1991). The 16S rRNA gene sequence from strain MM125-6 was obtained with a TA clone (vector pMD19T; TaKaRa). The complete 16S rRNA gene sequence was obtained from the draft genome using RNAmmer (Karin et al., 2007). The homology matrix of two 16S rRNA genes was analyzed using the DANMAN software (version 8.0). The 16S rRNA gene sequences of related type taxa were downloaded from the GenBank database. Similarities of the 16S rRNA gene sequence between the taxa were determined with the EzBioCloud 16S database.^4^ Based on 16S rRNA gene sequences, phylogenetic trees were constructed using the neighbor-joining (NJ) (Saitou and Nei, 1987), maximum likelihood (ML) (Felsenstein, 1981), and minimum evolution (ME) methods (Rzhetsky and Nei, 1992) of tree-making algorithms in MEGA Version 7 (Kumar et al., 2015). With the same software, Kimura’s two-parameter model was used to calculate the genetic distances for the above-mentioned analyses, and the bootstrap values were determined based on 1,000 replications.

Prior to genomic sequencing, the DNA integrity of *Alcanivorax* sp. strain MM125-6 was confirmed by agarose electrophoresis, and its concentration was measured with the Quantus Fluorometer (Picogreen). DNA libraries were generated with TruSeq DNA Sample Prep Kit (Illumina, Madison, WI, United States), and the genome was sequenced on an Illumina Hiseq 4000 platform using the paired-end protocol (2 × 150 bp). The reads were quality-controlled and filtered using Trimmomatic version 0.39^5^ as described in Bolger et al. (2014). The reads were checked for base call quality distribution. Furthermore, adapters and bases with Phred quality scores < 20 were trimmed from the right and left ends, and reads less than 25 bp were removed. A coverage of 378 × was achieved during the sequencing, and around 1,548.8 Mb of clean data (Q20 > 98%) were used to assemble the draft genome using megahit v 1.2.9 (Li D. et al., 2015) with option “-m 400 -t 96 -l 500.” CheckM was used to assess the completeness and contamination of the assembled genome for strain MM125-6 (Parks et al., 2015). The genome sequences of the three reference strains (*A. profundimaris* ST75FaO-1, *A. venustensis* ISO4, and *A. gelatiniphagus* MEBiC08158, based on the 16S rRNA gene phylogenetic tree) close to MM125-6 were downloaded from the GenBank database. The genomic DNA G + C content of strain MM125-6 was calculated based on its genome sequence. The genomes were uploaded to the RAST server and KEGG BlastKOALA^6^ (Kanehisa et al., 2016) for annotation^7^ (Aziz et al., 2008), and functional subsystem classifications were viewed using SEED Viewer version 2.0 (Aziz et al., 2008). After annotation, genes related to the autotrophy in the genomes of strain MM125-6 and the three strains were also analyzed by searching against the KEGG GENES database with BlastKOALA (Berg et al., 2010; Berg, 2011). To investigate the iron-related gene and gene neighborhood, the genomes of strain MM125-6 and the three strains were annotated with both tools of Fegenie^8^ and the hidden Markov models (HMMs) database (Garber et al., 2020).

### Carbon fixation ability test

For carbon fixation in the ^13^C isotopic tracer experiments, cells were grown in biological duplicates in 150-ml serum bottles containing 50 ml MMJHS medium prepared as described above, with 1 mM NaH^13^CO_3_ under a gas phase of 74% H_2_/20% CO_2_/6% O_2_ (200 kPa) in the headspace, and incubated at 28^°^C for 10 days. Before inoculation, cells in the exponential phase were centrifuged at 5, 000 rpm for 10 min, and the supernatant was discarded to remove the organic carbon carried in the inoculation solution. The pellet was washed with 1 × PBS at 500 rpm for 5 min and this was repeated thrice. The cell number of the inoculum under optimal conditions was around 3 × 10^8^ cells/ml. At a ratio of 1:100, 0.5 ml of the inoculum was added to 50 ml MMJHS medium. During incubation, 0.1 ml of the culture was sampled on days 1, 2, 3, 4, 6, 8, and 10 for cell counting and observed using a fluorescence microscope (Nikon 80i, Japan). Each sample was counted in five grids (containing 80 subgrids) of a hemocytometer under fluorescence microscopy and subsequently the results were extrapolated to calculate the cell concentration (Randolph, 1944). All the counts were done in duplicates. After sampling, the residue samples were centrifugated at 10,000 rpm for 10 min to collect the cell pellets. To remove the organic matter, all the pellets were washed thrice with 1 × PBS and further stored at −20^°^C. The pH of each sample was measured with a pH/ATC electrode (Sartorius, Germany). The pellets were lyophilized for 24 h and then sent to the Analysis and Testing Center at the Third Institute of Oceanography (Ministry of Natural Resources, Xiamen, China). δ^13^C isotopes of the cells were determined with the Stable Isotope Ratio Mass Spectrometer (IRMS) (measuring accuracy = ± 0.2‰; DeltaVAdvantage, Bremen, Germany). The procedures as described in Orcutt et al. (2015) were used to measure and calculate the δ^13^C ‰ of the cells.

### Iron oxidation ability assay

Agarose-based gradient tubes with different Fe(II)-containing substrates, such as energy sources, e.g., FeCO_3_, sulfide minerals, FeS, and zero-valent iron (ZVI), are commonly used to identify neutrophilic Fe(II)-oxidizing bacteria (Edwards et al., 2003b; Emerson and Floyd, 2005). This tool was used to test the Fe(II)-oxidation potential of strain MM125-6. The composition of a medium at the top and bottom of the gradient tubes is as described in Emerson and Moyer (2002), and contains 0.15% and 1% agarose (w/v), respectively. Fundamentally, an agarose semisolid medium in the gradient tube develops the oxic-anoxic transition zone with opposing gradients of oxygen and ferrous iron during oxygen descent from the air-medium interface downward and ferrous iron ascent from mineral medium upward. Fresh ZVI and hydrothermal massive sulfide powder can provide a Fe(II) source and electron for the top medium, and NaHCO_3_ can act as a sole carbon source for cell growth. To remove the organic matter in the cell suspension, 1 × PBS (NaCl 8 g, KCl 0.2 g, Na_2_HPO_4_ 1.44 g, and KH_2_PO_4_ 0.24 g) was utilized to wash cells 2∼3 times at 3,500 rpm for 20 min. After that, the tubes were inoculated vertically with a 0.1 ml pipette through the top medium, and a 0.3 ml cell suspension was injected at an interface between the top and bottom of the medium. The experiments were performed in an incubator at 28^°^C for 14 days. Growth well cells were stained with SYTO9 dye and observed by fluorescence microscopy (refer to below). The brown band in the gradient tube was collected and then centrifuged at 8,000 rpm for 20 min, and the supernatant was finally discarded. The bottom precipitation matter was stored at 4^°^C before SEM-EDS analysis.

### Fluorescence microscopy and scanning electron microscopy

To preserve cell activity and integrity, the enrichment slab was stored in a falcon tube filled with 10% glycerol TE stock and stored at −20^°^C prior to observation by fluorescent microscopy. Cells on the surface of the *in situ* enrichment slab and from the lab enrichment culture and pure culture were stained with LIVE/DEAD^®^ BacLight™ Bacterial Viability Kit (Thermo Fisher Scientific), which contained SYTO9^®^ and propidium iodide (Berney et al., 2007), and then observed or/and counted with a fluorescence microscope (Eclipse 80i, Nikon).

All of the samples were gradually dehydrated by washing with a graded series of 50, 70, 90, and 100% ethanol for 5 min each, and finally CO_2_-dried for 15 min. To prevent deformation and collapse of surface structure and to conserve surface morphology, the samples were lyophilized for 24 h under vacuum conditions. After mounting the samples on specimen holders, they were sputter-coated with platinum in a coating unit for approximately 2–3 min. The morphology and chemical composition of the samples were examined with a Phenom World Phenom Pharos Nano G2 scanning electron microscopy (SEM, Shanghai, China) device equipped with an energy dispersive spectrum (EDS, Phenom).

## Results

### Microbial community composition

**FIGURE 1:**
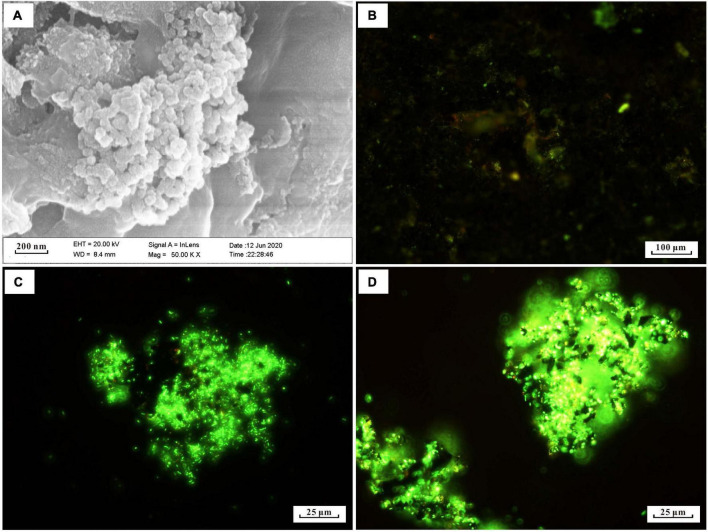
Microscopy images of biomineral structures characteristic and SYTO 9-stained cells of *in situ* enrichment slab surface and enrichment culture incubated with modified MMJHS medium, **(A)** SEM image of biomineralization with short stalk and cocci structure in slab. The SEM observation was performed at 20 kV with a working distance of 8.4 mm. SYTO 9-stained cells in **(B)** *in situ* enrichment slab; **(C)** original enrichment culture, and **(D)** secondary enrichment culture. Scale bars: **(A)** 200 nm; **(B)** 100 μm; **(C,D)** 25 μm.

Many short-stalked and cocci structures on the surface of the incubated slab were observed by SEM (Figure 1A). The EDS analysis identified these structures as amorphous particulate iron oxides (data not shown). To obtain information on the distribution of microbe cells on the incubated sample surface, we stained cells on the sample with the SYTO 9 dye. The fluorescent microscopy showed that there was an abundance of cells distributed on the sample’s surface (Figure 1B). Even though active cell and iron oxide were observed in the *in situ* enrichment slab, the specific microbes like FeOB cannot be determined with this method.

The number of cells in a preserved mixture that was washed from the surface of the *in situ* enrichment sample was nearly ∼3.93 × 10^5^ cells/ml. After the primary laboratory culture (inoculation ratio is 1:100), the cell density in the JL125_P_1 and JL125_P_2 samples was increased from ∼10^3^ cells/ml at 0 h to up to at least ∼10^6^ cells/ml at the end of the incubation (many cells stuck on the sulfide powder tightly, Figure 1C). The exponential-phase cells of the primary enrichment culture (JL125_02) were inoculated into the medium for subculture. The cell density was initially around 2.95 × 10^4^ cells/ml and was increased up to 3.75 × 10^6^ cells/ml at the end of the enrichment, excluding a part of cells that was attached to the sulfide powder (Figure 1D). Thus, the modified MMJHS medium was suitable for cell growth.

**FIGURE 2:**
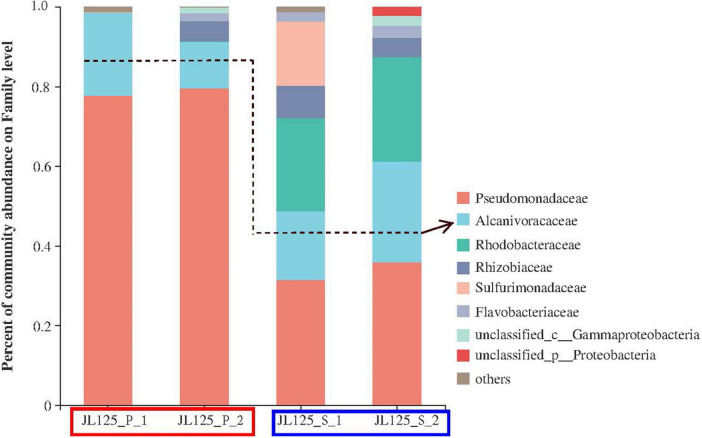
Relative abundance of the active bacterial community based on the frequency of bacterial 16S rRNA gene in clone libraries at primary (red frame) and secondary (blue frame) enrichment cultures with modified MMJHS medium. The surface biofilms on the *in situ* sample were inoculated to the modified MMJHS medium with hydrothermal massive sulfide powder as energy source. In order to exclude the influence of relic nucleic acid molecules in the initial inoculum and obtain a stable bacterial community, the primary cultures need to be inoculated into a new medium.

After the enrichment, cell DNA was extracted from the enrichment cultures. A total of 66 OTUs were obtained and analyzed (i.e., 25 for JL125_P_1, 19 for JL125_P_2, 46 for JL125_S_1 and 25 for JL125_S_2 collected at the end of the enrichment; Supplementary Table 1). The clones in the library of the primary culture were affiliated with the following taxonomic groups (Figure 2): the active bacterial community was mainly composed of *Gammaproteobacteria* (92.66–98.95%), *Alphaproteobacteria* (0.004–5.27%), *Campylobacteria* (0–0.8%), *Actinobacteria* (0–0.39%), *Bacteroidia* (0–2.04%), and unclassified *proteobacteria*. Many of the phylotypes in the primary cultures are similar to the *in situ* enrichment clones recovered from the sulfide mineral of the active vents (Wang C. H. et al., 2020) and the uncultured environmental clones retrieved from the plumes of the active vents (Sylvan et al., 2012a). Simultaneously, these phylotypes were also detected in secondary enrichment cultures at the end of 1 month of long-term incubation (Figure 2). In the secondary enrichment cultures, the active bacterial communities were composed of *Pseudomonadaceae* (31.40–35.92%), *Rhodobacteraceae* (23.39–26.22%), *Alcanivoracaceae* (17.31–25.21%), and *Sulfurimonadaceae* (0–16.14%). Some of them were inferred to be involved in the deep-sea element cycle: *Pseudomonadaceae, Alcanivoraceae, Rhodobacteraceae*, and *Sulfurimonadaceae* were related to the sulfur cycle, and *Pseudomonadaceae, Alcanivoraceae, Rhodobacteraceae*, and *Rhizobiaceae* participated in the iron cycle (Smith et al., 2011; Callac et al., 2015; Percak-Dennett et al., 2017).

The dominant genera in the primary and secondary enrichment samples were *Pseudomonas*, *Alcanivorax*, and *Martelella* (Supplementary Figure 3). *Pseudomonas* ranged in relative abundance between 77.7 and 79.6% for the primary samples, and between 31.4 and 35.9% for the secondary samples. *Martelella* ranged between 0 and 5.1% for the primary samples, and between 4.9 and 8.1% for the secondary samples. *Paracoccus* was not dominant in the primary samples, but its relative abundance was increased to 23.4–25.6% in the secondary samples. The genus *Alcanivorax* belonging to the class *Gammaproteobacteria* was detected in both the primary and secondary enrichment cultures (Supplementary Figure 3). The percentage of the total number of clones for this genus in the four samples was 20.87% (JL125_P_1), 11.63% (JL125_P_2), 17.31% (JL125_S_1), and 25.21% (JL125_S_2). Nevertheless, the relative abundance of OTUs for this genus was less than 5% in the bottom seawater of the Wocan-1 site (Liu, 2020). *Alcanivorax* has a relatively higher abundance in all the enrichment cultures, probably indicating that they have an iron oxidation function and are closely associated with sulfide minerals during the incubation periods.

### Phylogenetic tree analysis of *Alcanivorax* sp. MM125-6

**FIGURE 3:**
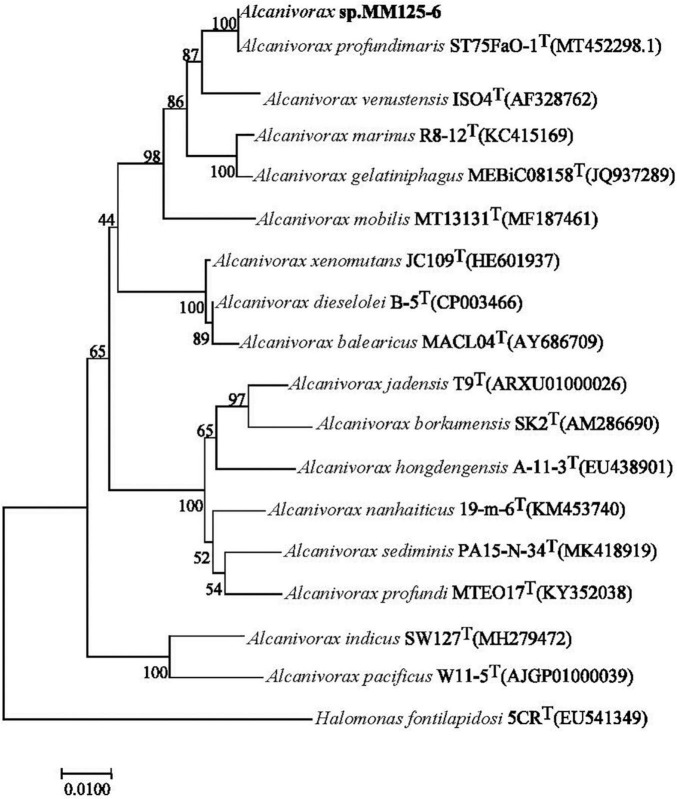
Neighbor-joining tree showing the phylogenetic positions of strain MM125-6 in the genus *Alcanivorax* based on full-length 16S rRNA gene sequence using MEGA 7. Bootstrap value represents 1,000 replicates and random seeding. *Halomonas fontilapidosi* 5CR*^T^* (EU541349) was used as outgroup.

Strain *Alcanivorax* sp. MM125-6 was isolated from the enrichment culture in the MMJHS medium. The length of the 16S rRNA gene sequence from PCR amplification was 1,407 bp, and its integrity was 96.8%. The full-length 16S rRNA gene (1,546 bp) of strain MM125-6 was obtained from the draft genome sequence with the RAST server. The DNAMAN software (version 8.0) analysis suggested that the homology of the two sequences was 99.8%. The phylogenetic analysis based on the full-length 16S rRNA gene showed that strain MM125-6 belonged to the genus *Alcanivorax* and shared 98.9% similarity with closet-type strain *Alcanivorax profundimaris* ST75FaO-1*^T^*, which was isolated from the epipelagic seawater of South China Sea (Dong et al., 2021). Meanwhile, it shared phylogenetic similarities with some members of the *Alcanivorax* (Figure 3) genus, such as *A. venustensis* ISO4*^T^* (98.08%), *A. marinus* R8-12*^T^* (97.88%), *A. gelatiniphagus* MEBiC08158*^T^* (97.66%), *A. mobilis* MT13131*^T^* (96.44%), *A. dieselolei* B-5*^T^* (96.17%), *A. xenomutans* JC109*^T^* (96.00%), and *A. balearicus* MACL04*^T^* (95.69%), and for the other species, it shared a < 95% sequence similarity. These strains were isolated from various marine environments where the salinity was nearly 35 g/L and their limited NaCl for growth could be higher than 40 g/L, indicating that they could adapt to high salinity conditions.

### General genome features and functional annotation

Over 1.59 Gb of paired-end reads were generated from the genome sequencing of *Alcanivorax* sp. strain MM125-6 with the Illumina Hiseq 4000 sequencing technology and subsequently assembled using megahit version 1.2.9, which has an average G + C content of 66.13 mol% (Supplementary Tables 2, 3). The analysis of the draft genome in CheckM (version 1.1.6) revealed that the complete and the contaminated were 100 and 0.2323%. The resulting assembly genome consists of 124 contigs of over 500 bp (*N*_50_, 154,497 bp; maximum contig size, 515,527 bp) totaling to 4.1 Mb, as well as 47 tRNA and 8 ribosomal RNA operons (Supplementary Table 3). The automatic genome annotation using the RAST server (Aziz et al., 2008), based on megahit v 1.2.9 assembly genomic contigs, gave a 31% subsystem coverage for 3,827 putative coding sequences (Supplementary Table 3). There was a higher abundance of genes for amino acids and their derivatives, carbohydrates, cofactors, vitamins, prosthetic groups, pigments, fatty acids, lipids, isoprenoids, and protein metabolism with the RAST annotated subsystem (Supplementary Figure 4).

We investigated the genome of MM125-6 to determine genes involved in autotrophy, including possible metabolic pathways for carbon fixation (Supplementary Figure 5). The KEGG BlastKOALA annotation results showed that most known genes involved in the CBB cycle were confirmed in the genome of MM125-6 except for phosphoribulokinase, fructose-1,6-bisphosphatase class 2/sedoheputulose-1,7-bisphosphatase, and ribulose-1,5-bisphosphate carboxylase/oxygenase (Rubisco). In addition, it also lacks key enzymes related to the rTCA cycle, including genes for 2-oxoglutarate synthase, ATP citrate lyase (ACL), and citryl-CoA synthetase (CCS)/citryl-CoA lyase, which may influence the operation of the reductive TCA cycle (Supplementary Figure 5). The lack of genes for CBB and rTCA pathways for carbon fixation is also observed in the genome of *A. profundimaris* ST75FaO-1, *A. venustensis* ISO4, and *A. gelatiniphagus* MEBiC08158 (results not shown).

To determine genes that may be involved in Fe(II) oxidation, the genome sequence of strain MM125-6 was annotated with Fegenie tools. The results showed that iron-related metabolic genes that are involved in iron transport, heme transport, siderophore transport, iron gene regulation, and iron storage were found in the genome (Supplementary Figure 6). Three putative proteins that are homologs of Fe oxidase cyc1 have also been identified in the genome of strain MM125-6, *A. profundimaris* ST75FaO-1, *A. venustensis* ISO4, and *A. gelatiniphagus* MEBiC08158 (Supplementary Table 4). All of the four strains lack putative proteins homologous to the cyc2 and rusticyanin from *Acidothiobacillus ferrooxidans* (Castelle et al., 2008). Aside from strain MM125-6, the presence of the FoxY homolog, which belongs to the proposed photosynthetic Fe(II) oxidation system FoxEYZ (Croal et al., 2007), was confirmed in the other three strains.

### Carbon fixation by ^13^C-enrichment of NaH^13^CO_3_

**FIGURE 4:**
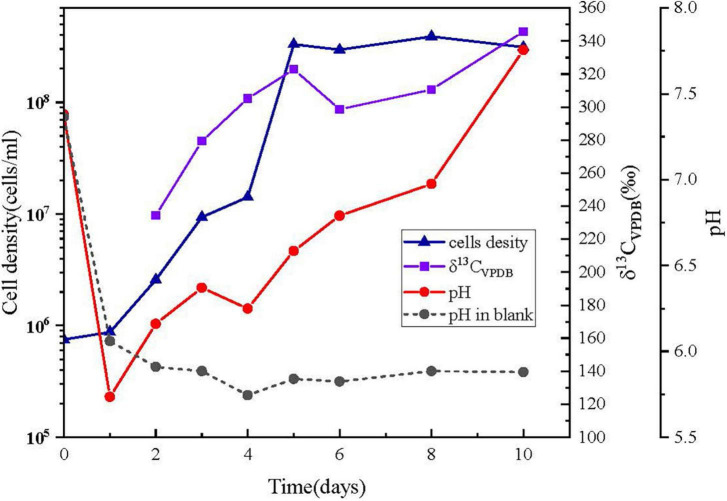
Changing features of cells number, pH, and δ^13^ C_VPDB_ (‰) in cells with incubation time. (▲) cell numbers; (■) δ^13^ C_VPDB_ (‰) incubation cells was too less to measure the ^13^C at day 1; (•) pH, symbols with solid line and short dash represent the inoculums and blank experiment, respectively.

The cells of *Alcanivorax* sp. MM125-6 was inoculated into the MMJHS medium and cultured for 10 days at 28^°^C. The fluorescent microscopy observation of cells stained with SYTO9 showed an increased concentration of active cells on day 10 compared to on day 2 (Supplementary Figure 7). The changing features of cell densities and pH are shown in Figure 4. The cell density of the incubation was increased from 7.5 × 10^5^ cells/ml at 0 h to up to 3 × 10^8^ cells/ml on day 5 and then maintained steadily until the end of the experiment (Figure 4). Therefore, the increase in cell numbers indicated that the inorganic MMJHS medium was suitable for strain MM125-6 growth.

The initial pH value of the experiment was 7.38 ± 0.02, but it was decreased to 5.74 ± 0.02 within 24 h and finally was increased up to 7.76 ± 0.02 on day 10 in the inoculum experiments. Conversely, the pH value was decreased to 6.06 ± 0.02 on day 1 and remained at 5.70–6.00 on days 2–10 in the control experiment. The pH drop in 1 day might be a result of the dissolution of CO_2_ from the headspace. However, the changes in pH were different when microbes were present and absent, probably due to cellular metabolism producing alkaline substances in the system. For example, cells might oxidize thiosulfate to intermediate tetrathionate, leading to increase in pH in the system, which was also found in *Pseudomonas* and *Halomonas* species (Sorokin et al., 1999; Teske et al., 2000; Sorokin, 2003).

The labeled ^13^C isotopes of NaH^13^CO_3_ in the liquid medium were used as a carbon source for cell growth. The accumulation of ^13^C labeled from bicarbonate in the MM125-6 biomass was 234.25‰ (δ^13^C_VPDB_) on day 2, and it was gradually increased up to 345.66‰ by day 10 (Figure 4). The results suggested that the inorganic carbon in NaH^13^CO_3_ can be used by strain MM125-6.

### Iron oxidation

**FIGURE 5:**
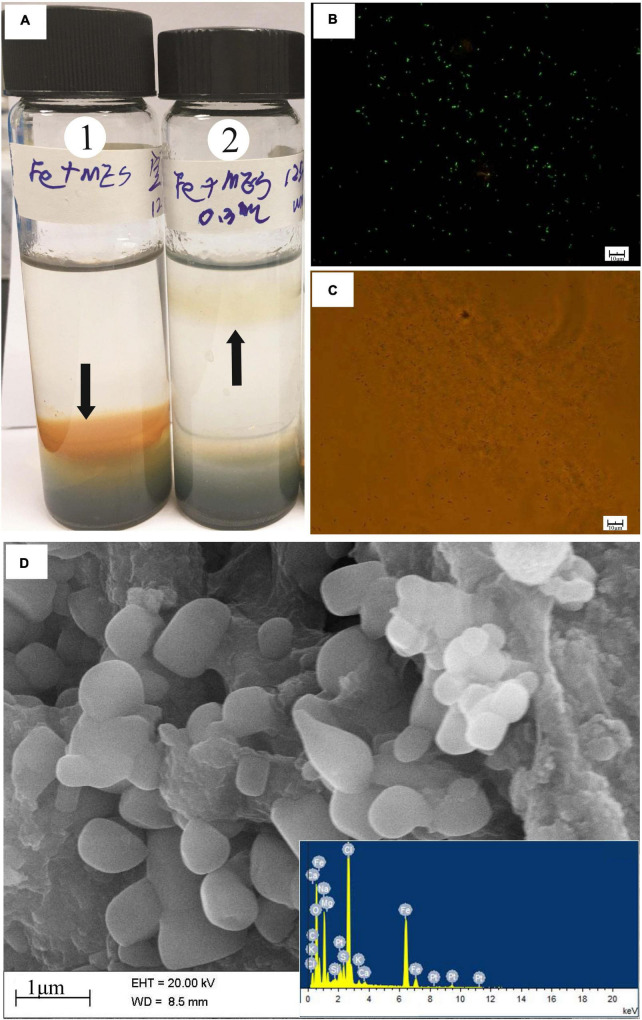
**(A)** O_2_/ZVI gradient tubes incubated for 14 days at 28°C. Tube 1 is an uninoculated control; tube 2 is inoculated with 0.3 ml of strain MM120-6. In the culture tube, discrete bands of microbes growth ∼1.5 cm off from gas-medium interface at the top of the tube. Black arrows indicate the position of the microbe growth region and iron oxides in the tubes. The milky band contains abundant cells observed by microscopy in fluorescent and brightfield **(B,C)**. SEM and EDS images of iron oxides band in the biotic experiment **(D)**. In the abiotic control, iron oxides develop at ∼0.3 cm above the top-bottom medium interface at the top. The SEM observation was performed at 20 kV with a working distance of 8.5 mm. Scale bar: **(B,C)** 10 μm; **(D)** 1 μm.

Isolate strain MM125-6 was tested for Fe(II) oxidation in gradient tubes and positively showed Fe(II) oxidation with either ZVI (Figure 5) or hydrothermal massive sulfide (results not shown) as the Fe(II) source as compared to the abiotic control. Discrete zones displaying brown and reddish-brown zones of iron oxide precipitates were developed in biotic and abiotic experiments after 14 days (Figure 5). In the gradient tubes, cell bands formed approximately 1.5 cm below the air-agarose medium interface. The cell bands were overlain by an opaque region that was rich in iron oxide precipitates (Figure 5A). The position of iron oxides produced in the abiotic tubes was distinct from that in the culture experiment, which was located at the interface of the top-bottom medium, and the iron oxides had a deeper brown color. Meanwhile, the position of cell bands in the gradient tubes in our ZVI and hydrothermal massive sulfide powder experiment (data not shown) resembles that in the synthetic ferrous sulfide experiment (Rhine et al., 2008). The fluorescent microscopy showed abundant cells in a milky band (Figures 5B,C) where they do not produce biogenically templated Fe(III) structures, such as twisted stalks and tubular sheaths. Similarly, strain *Alcanivorax sp*. 3B.1 isolated from *in situ* enrichment minerals in the ocean crust was proven to be able to oxidize Fe(II) (Smith et al., 2011).

SEM-EDS images of the brown band substance incubated for 14 days in the presence of *Alcanivorax* sp. strain MM125-6 was presented in Figure 5D. The SEM-EDS images showed that there was an abundance of ellipsoidal iron oxides, which were mainly composed of C, O, Fe, Na, and Cl, in the brown band (Figure 5D). This substance had a high percentage of oxygen atoms (48.6%), which signified that the environment where the brown band was located was in an oxidation state. Excluding sodium chloride content (15.2% atomic percentage), the brown band substance was composed of organic carbon and iron oxide. Although the type of the Fe(III)-bearing mineral was not determined in this study, based on the above results, we tentatively draw the conclusion that *Alcanivorax* sp. MM125-6 has a capacity for autotrophic Fe(II)-oxidation.

### Strain MM125-6 interacted with hydrothermal massive sulfide

**FIGURE 6:**
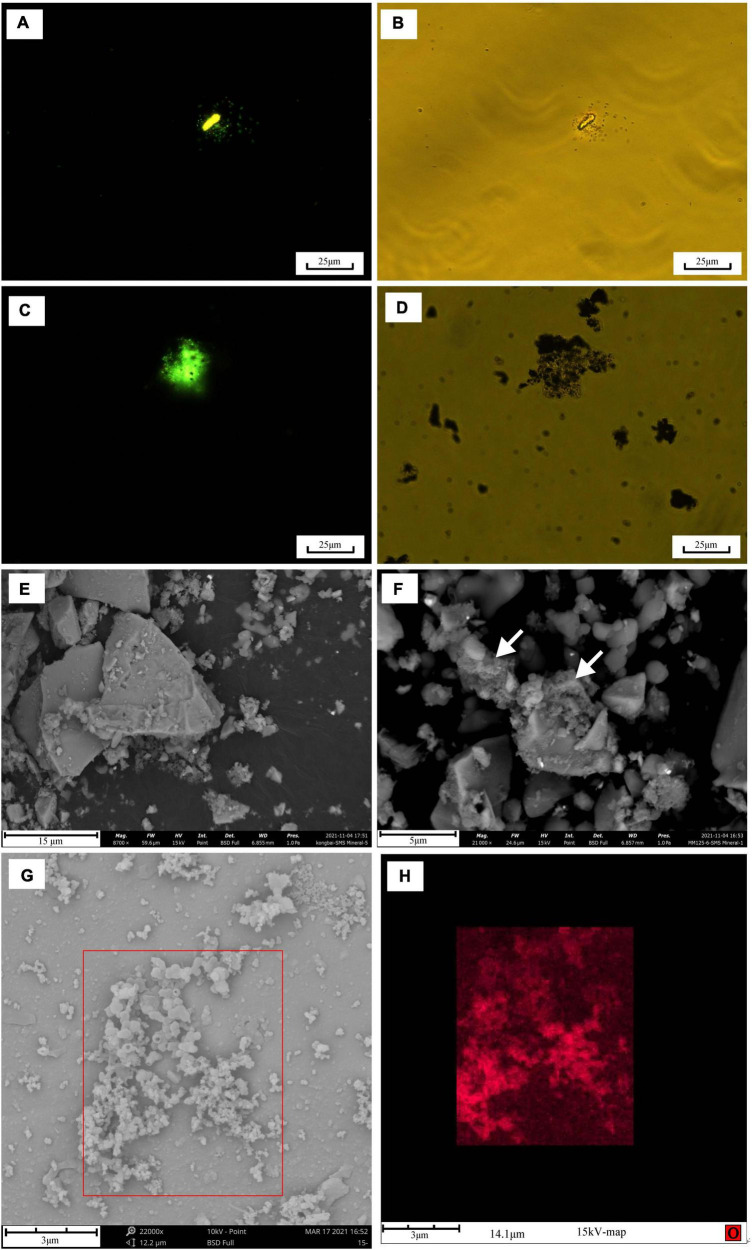
Microscopy images of SYTO 9-stained cells grown in the presence of hydrothermal sulfide for 1 month in the modified MMJHS medium during and after the experiment and of morphology changes on the surface of hydrothermal sulfide. Fluorescent microscopy and corresponding brightfield images of incubation cells: **(A,B)** at day 5; **(C,D)** at 1 month. SEM images of hydrothermal sulfide morphology in abiotic **(E)** and biotic **(F)** experiment. In **(F)** cotton-like floes are visible on the surface (white arrows). SEM BSD image of biogenetic iron oxides on the surface of hydrothermal sulfides **(G)** and EDS map of oxygen element **(H)** corresponding to the boxed region of **(G)**. The operation parameters of SEM during observation are respectively shown at the bottom of the above SEM pictures. In **(H)**, oxygen in the particulate iron oxides appears in red against the background mineral (metal sulfide). Scale bars: **(A–D)** 25 μm; **(E)** 15 μm; **(F)** 5 μm, and **(G,H)** 3 μm.

We transferred *Alcanivorax* sp. MM125-6 to oligotrophic conditions in a basic mineral medium with hydrothermal sulfide and NaHCO_3_ and without headspace gas. The inoculum was a pure culture growing under chemolithoautotrophic conditions in an MMJHS medium. The geochemistry analysis showed that the Fe (II)/TFe ratio of the initial medium was lower than 0.071 because of the content of Fe(II) and TFe (total Fe) was scarce in the background seawater. The Fe (II)/TFe ratio was in the range of 0.466–0.798 in the inoculated cultures and was equal to 0.301 for the abiotic control, suggesting that the iron oxidation function of cells would contribute to the weathering of hydrothermal sulfide (data unpublished). This may be ascribed to either the rate of Fe(II) release from the sulfide is faster than the oxidation rate or that there is simply more Fe(II) available than cells are able to process (Edwards et al., 2003b). Fluorescent microscopy was performed on incubation cells mixed with powder sulfide. Fluorescent microscopy and bright field images showed that minerals were surrounded by low numbers of activity cells on day 5 (Figures 6A,B). At 1 month, abundant activity cells were attached to the surface of the mineral (Figures 6C,D), which implied that the concentration of cells had increased qualitatively compared to on day 5. This may suggest that MM125-6 can oxidize the Fe(II) from sulfide minerals to release energy that can be harnessed for cellular processes.

SEM-EDS images of the surface of hydrothermal massive sulfide incubated for 3 months in the presence of *Alcanivorax* sp. MM125-6 are presented in Figure 6. We also imaged an abiotic control as a contrast for our biotic enrichments (Figure 6E). The SEM image showed that the sulfide mineral surface reacted in the abiotic experiments was relatively clean with only very few iron oxide particles deposited in the abiotic experiment (Figure 6E). In contrast to abiotic reactors, the SEM image indicated that abundant cotton-like materials mixed with iron oxides were covered on the surface of sulfide minerals in the biotic culture (Figure 6F). These materials are very similar in appearance to exopolysaccharides structures that have recently been recognized as specific biomolecules that act on sulfide minerals to promote dissolution (Edwards, 2004; Sievert and Vetriani, 2012). Amorphous iron oxides appeared with a particulate shape, and biominerals (hollow interior) with curious morphology were observed on the surface of hydrothermal massive sulfides (Figure 6G). EDS map data revealed that the structures were iron oxides, which constitute Fe, S, O, C, and P (data not shown). Compared to the background minerals, oxygen signal was intense in the iron oxides, which indicated that Fe(II) oxidation occurred on the surface of hydrothermal massive sulfides (Figure 6H). Overall, these results demonstrate that cells are obviously attached tightly to the surface of sulfide minerals, which indicates direct interactions between MM125-6 and sulfide minerals.

## Discussion

Many short-stalked and cocci structures were observed on the surface of the *in situ* enrichment hydrothermal massive sulfide slab (Figure 1), which was similar to those of biogenic iron oxides in the hydrothermal field (Edwards et al., 2003a). This suggested that iron-oxidizing bacteria were active on the mineral surface. According to the microbial analysis with molecular methods, sulfur- and iron- oxidizers belonging to *Gamma*-, *Alpha*-, and *Beta-proteobacteria* are considered to be key players in sulfur and iron metabolism that probably corresponds to sulfide mineral weathering in deep-sea hydrothermal fields (Wirsen et al., 1993; Edwards et al., 2003a; Suzuki et al., 2004; Kato et al., 2017; Van Dover, 2019; Hou et al., 2020). However, comprehensive understanding of microbial phylotypes and culturable isolates and their metabolism characteristics inhabited in sulfide minerals in deep-sea hydrothermal fields is still lacking.

The use of hydrothermal sulfide as an energy source is conducive to enrichment of iron- or sulfur- oxidizing bacteria under conditions similar to those in marine environments. Previous experimental studies performed under aerobic conditions at 4^°^C using a medium made of artificial seawater and seafloor massive sulfide slabs have reported bacterial phylotypes related to *Halomonas*, *Marinobacter*, *Methylophaga*, and *Pseudomonas* (Kato et al., 2015). The dominant genus *Marinobacter*, involved in iron oxidation (Edwards et al., 2003b), and the genus *Halomonas*, affiliated with acid-producing bacteria (Sanchez-Porro et al., 2010), were both adhered to the slabs, which probably contribute to release of Zn from sulfide minerals. In our laboratory enrichment, we found that three genera, *Pseudomonas, Paracoccus*, and *Alcanivorax* from the slab were effectively enriched and eventually attached to the metal sulfide powder (Figures 1, 2), indicating their potential for iron or sulfur oxidation and interaction between cells and sulfide minerals (Napieralski et al., 2021).

In our enrichment, the genus *Pseudomonas* belong to the *Pseudomonadaceae* family was dominant in the taxonomic groups of all the samples. This genus has been detected in various marine environments, such as basaltic rock (Bailey et al., 2009; Sudek et al., 2009), active submarine volcanoes (Bravakos et al., 2021), and hydrothermal fields (Wang et al., 2002; Hou et al., 2020). In this genus, many isolates from marine environments were tested and recognized as siderophore-producing bacteria (Sudek et al., 2009; Perez et al., 2019), heterotrophic iron oxidizers (Smith et al., 2011; Rowe et al., 2014; Sudek et al., 2017), and nitrate reducers (Straub et al., 1996; Bedzyk et al., 1999; Busquets et al., 2013; Pena et al., 2013). They have been shown to be capable of growth on a variety of synthetic mineral substrates, such as FeCO_3_ and FeSO_4_, as their sole energy source under anaerobically in the presence of nitrate (Straub et al., 1996; Smith et al., 2011). Genome analysis of 20 strains of the genus *Pseudomonas* stutzeri demonstrated that complete sets of genes both for denitrification and nitrogen fixation pathways were detected in their genomes (Busquets et al., 2013; Pena et al., 2013). More recently, the accumulation of convincing data from the laboratory suggested that the *Pseudomonas* genus was the specific microbe responsible for submarine basalt bioalteration (Bailey et al., 2009; Sudek et al., 2009; Smith et al., 2011; Chen et al., 2014). It can utilize structural Fe(II) in basaltic glass as its energy source for growth. For example, strain *Pseudomonas stutzeri* VS-10, a metabolically versatile bacterium isolated from the Vailulu’u seamount, can form biofilms and encrust filaments on basaltic glass to accelerate Fe(II) oxidation under heterotrophic conditions (Sudek et al., 2017). Our results augment the hypothesis that the *Pseudomonas* genus can oxidize Fe(II) and play a role in iron-rich substrate weathering in deep sea environments. Overall, this genus is metabolically and functionally versatile, which supports their adaptability to extreme deep sea environments where nutrients are oligotrophic.

The *Paracoccus* in genus the *Rhodobacteraceae* family is the second most abundant genera in the two secondary enrichment cultures. The *Paracoccus* genus has been widely recovered from marine and terrestrial environments (Yoon et al., 2019). Some *Paracoccus* strains are facultatively lithoautotrophic and neutrophilic bacteria that can grow using various compounds and inorganic electron donors, such as molecular hydrogen or thiosulfate (Friedrich, 1997; Friedrich et al., 2000; Ghosh and Roy, 2007; Ramasamy et al., 2020). Additionally, pure cultures of *P. denitrificans* (Muehe et al., 2009), *P. ferrooxidans* (Kumaraswamy et al., 2006), and *P. pantotrophus* (Price et al., 2018) have demonstrated the capability to oxidize Fe(II) with nitrate by potential denitrification pathways. Thus, the dissolved sulfur compounds and Fe(II) from sulfide minerals may be oxidized by this group for cell growth during incubation time.

In our enrichment results, the *Alcanivorax* genus was the third dominant bacterial group, and its relative abundance remained consistent in the primary and secondary enrichment cultures. The *Alcanivorax* genus is known to be widely distributed in marine environments, e.g., hydrothermal vent plumes (Wang W. et al., 2020), hydrothermal fluids (Bertrand et al., 2013), and crustal fluids (Orcutt et al., 2020). They have previously been deemed to obligate marine hydrocarbon decomposers that can degrade a wide spectrum of straight and branched alkanes (Yakimov et al., 1998, 2019; Dong et al., 2021; Sinha et al., 2021). Recently, they have also been detected in Fe(II)-bearing minerals incubated at oceanic crust boreholes that are influenced by ambient water flow and crustal fluids (Smith et al., 2011; Orcutt et al., 2020). Combined with our results, we suspect that *Alcanivorax* originated from seawater and then colonized on the iron-rich mineral surface as a successor population. Nonetheless, to the best of our knowledge, we are unaware of any research analyzing how the *Alcanivorax* genus can thrive on the iron-rich mineral surface. Intriguingly, the 3B.1 strain isolated from the surface of a basalt slice in an oceanic crust had first been sorted as an iron oxidizer in the genus *Alcanivorax* (Smith et al., 2011). Two strains (YD13-A and YDC2-H) in the *Alcanivorax* genus were isolated from hydrothermal sediments using NaHCO_3_ or CO_2_ as the carbon source (Liu et al., 2009), implying their autotrophic carbon fixation function. These data, together with the observation of high enrichment of the *Alcanivorax* genus in the modified MMJHS medium, strongly suggest that this genus must be a potential autotrophic iron oxidizer.

The *Alcanivorax* sp. strain MM125-6 was isolated from an *in situ* enrichment slab using an oligotrophic MMJHS medium by the dilution-to-extinction method in this study. The oligotrophic MMJHS medium was typically designed to incubate or isolate chemoautotrophic microbes that can fix carbon dioxide (Inagaki et al., 2003), such as the genera *Sulfurimonas*, *Sulfovum*, *Nitratiruptor*, and *Desulfothermus* (Nakagawa, 2005; Takai et al., 2006; Nunoura et al., 2007). Although the *Alcanivorax* sp. YD13-A and YDC2-H strains were first isolated from the MMJHS medium (Liu et al., 2009), the chemoautotrophic function for the *Alcanivorax* genus was not reported. In our study, strain MM125-6 can grow well with NaHCO_3_ as the carbon source, and its cell concentrations can increase up to 3.3 × 10^8^ cells/ml (Figure 4 and Supplementary Figure 7). Therefore, we can conclude that MM125-6 is the first strain in the *Alcanivorax* genus that can grow autotrophically.

In addition, strain MM125-6 could grow well with Fe(II) and hydrothermal massive sulfide as electron donors and form iron oxide bands near the top of the interface medium (Figure 5). The gradient tube is the most common and effective method to enrich, isolate, and identify neutrophilic Fe(II)-oxidizing bacteria. In the gradient tube, oxygen from the headspace and ferrous iron from the bottom will occur in biogeochemical reactions at specific locations where Fe(II)-oxidizing bacteria thrive (Emerson and Moyer, 1997; Edwards et al., 2003b). Fe(II) oxidation and NaHCO_3_ in the semi-solid medium act as energy source and sole carbon source for cell biosynthesis, respectively (Emerson and Moyer, 2002; Emerson and Floyd, 2005). Many Fe(II)-oxidizing bacteria have been isolated and identified by gradient tubes, including but not limited to the chemoautotrophic *Mariprofundus ferrooxydans* genus (reviewed in reference McAllister et al., 2019), chemoheterotrophic *Marinobacter* (Edwards et al., 2003b; Smith et al., 2011; Müller et al., 2014; Leslie et al., 2015), and strains belonging to *Hyphomonas jannaschiana* (Edwards et al., 2003b). One strain (3B.1) affiliated to the *Alcanivorax* genus isolated from *in situ* enrichment igneous minerals and tested with gradient tubes was able to oxidize Fe(II) heterotrophically (Smith et al., 2011). Intriguingly, cultivating experiments in the modified MMJHS medium, with NaHCO_3_ and hydrothermal sulfide minerals, have attested that MM125-6 can excrete the acidic exopolysaccharides, which facilitate cell attachment to the surface of sulfide minerals (Figure 6). Iron oxide particles mixed with exopolysaccharides were observed frequently encrusting sulfide minerals by SEM (Figures 6F,G). EDS maps of oxygen showed that elements of oxygen mainly exist in oxides but are scarce in background minerals (Figure 6H). These element compositions were akin to the product shapes of pyrite oxide mediated by iron-oxidizing bacteria, such as *Acidithiobacillus ferrooxidans* (Li J. et al., 2015) and *Leptospirillum ferrooxidans* (Harneit et al., 2006), and further indicated the iron oxidation function of MM125-6. Together, the functions of autotrophic, iron oxidation and production of exopolysaccharides enable us to explain why the ability of strain MM125-6 grows in oligotrophic modified MMJHS medium and how the genus *Alcanivorax* adheres to and is active on the surface of iron-rich substrates in various deep sea environments.

Previous studies have shown that Calvin-Benson-Bassham (CBB) is a common carbon fixation pathway for iron oxidizers the deep-sea hydrothermal vents, such as *Zetaproteobacteria* (McAllister et al., 2019; Mcallister et al., 2020) and *Gammaproteobacteria* (Hügler and Sievert, 2010; Jan et al., 2014; Hou et al., 2020). Additionally, the genes of several free-living strains belonging to the subdivision of the *Gammaproteobacteria* class have encoded both the rTCA and CBB cycles (Anantharaman et al., 2016; Rubin-Blum et al., 2019). Several known genes involved in the CBB cycle were not found in the draft genome of strain MM125-6 (Supplementary Table 4), and key enzymes concerning the rTCA cycle were also lacking (Sutherland et al., 2017), which may influence the operation of CBB and reductive TCA cycle (Buchanan and Arnon, 1990). The lack of genes for CO_2_ fixation has also been observed in other *Alcanivorax* members (data not shown). This may preliminarily suggest that strain MM125-6 or genus *Alcanivorax* is unable to use the CBB or rTCA pathway to fix carbon because of the absence of key enzymes. Additionally, the annotation results revealed the presence of iron-related metabolism genes in the genome (Supplementary Figure 6), indicating the important role of strain MM125-6 in iron cycling in deep-sea hydrothermal environments. Homologs of the Fe oxidase *Cyc1* have been annotated in the genome of strain MM125-6, *A. profundimaris* ST75FaO-1, *A. venustensis* ISO4, and *A. gelatiniphagus* MEBiC08158 (Supplementary Table 4). Nevertheless, other dedicated ferroxidases that are involved in the enzymatic Fe(II) oxidation pathway for known iron-oxidizers were not found in the genome of the above four strains, such as *Cyc2*, *Cyt572*, *Cyt579* (Tan et al., 2019; Mcallister et al., 2020), MtoAB (He et al., 2017), and PioABC (Jiao and Newman, 2007). Further investigations to determine the carbon fixation and Fe(II) oxidation pathways of genus *Alcanivorax* should be combined with genomic, enzymatic, transcriptomic, and proteomic approaches.

## Conclusion

Since the steep redox gradient between the deep-sea hydrothermal sulfide and seawater is where microorganisms live in, it is feasible to assume that their features and components might breed the richness of chemoautotrophic or chemoheterotrophic iron-oxidizing bacteria. The *Alcanivorax* genus from the surface of the *in situ* enrichment slab was effectively enriched using the modified MMJHS medium with metal sulfide powders, indicating its potential for autotrophic carbon fixation and iron oxidation. Unfortunately, in addition to hydrocarbon degradation, other metabolic potentials like carbon fixation and iron oxidation of the *Alcanivorax* genus in deep-sea hydrothermal fields have received less attention. Here, strain *Alcanivorax* sp. MM125-6 was isolated from the *in situ* enrichment hydrothermal massive sulfide slab with the MMJHS medium using the dilute-to-extinct method. Increase in cell number and accumulation of ^13^C in cells in the MMJHS medium with NaH^13^CO_3_ as the carbon source was both observed. Additionally, we found a brown and reddish-brown zone of iron oxides and cell band near the top of the O_2_/ZVI gradient tube. Furthermore, it can also secrete acidic exopolysaccharides and adhere to the surface of sulfide minerals to oxidize Fe(II), which accelerates sulfide dissolution. One possible explanation for these observations is that strain MM125-6 is capable of autotrophic carbon fixation and chemoautotrophic Fe(II) oxidation. These metabolic functions probably favor the *Alcanivorax* genus adapting to various marine environments, especially iron-rich substrates. Based on our knowledge, this is the first specific study on *Alcanivorax* spp. from a hydrothermal vent, with autotrophic carbon fixation and Fe(II)-oxidation functions. However, further analyses are required to identify the metabolic pathways of carbon fixation and Fe(II)-oxidation for the *Alcanivorax* genus in the presence of hydrothermal massive sulfide as well as the interplay between them in deep-sea hydrothermal fields.

## Data availability statement

The datasets presented in this study can be found in online repositories. The names of the repository/repositories and accession number(s) can be found in the article/Supplementary material.

## Author contributions

MW performed the experiments and wrote the manuscript. XZ and XH designed the research and analyzed the data. ZS provided the platform and guidance for the experiment. YW and ZQ executed the DY49th and DY57th cruises as well as deployed and retrieved the *in situ* hydrothermal sulfide samples. CD and QX carried out fluorescence microscopy and electron microscopy observations. All authors edited the manuscript, contributed to the article, and approved the submitted version.

## Footnotes

1
https://lpsn.dsmz.de/genus/alcanivorax

2
https://report.majorbio.com/meta/

3
http://sourceforge.net/projects/rdp-classifier

4
https://www.ezbiocloud.net/

5
http://www.usadellab.org/cms/index.php?page=trimmomatic

6
https://www.kegg.jp/blastkoala/

7
https://rast.nmpdr.org/

8
https://github.com/Arkadiy-Garber/FeGenie
