# Supplementary material for: Potential autotrophic carbon-fixer and Fe(II)-oxidizer Alcanivorax sp. MM125-6 isolated from Wocan hydrothermal field
Source: Front Microbiol. 2022 Oct 14;13:930601. doi: 10.3389/fmicb.2022.930601 (PMC9616709; doi:10.3389/fmicb.2022.930601)
Supplement: Supplementary file 1 [file Data_Sheet_1.zip › Tables S1 - S3.DOCX]

| **Supplementary Table S1.** Analysis of the bacterial diversity from 16S rRNA gene libraries in enrichment culture of original and subculture. | | | | | | |
| --- | --- | --- | --- | --- | --- | --- |
| Sample | Number of OTUs  (> 97% similarity) | Coverage | ACE | Chao1 | Shannon  index | Invsimpson  indice |
| JL125_P_1 | 25 | 1.00 | 29.55 | 27.50 | 0.64 | 1.56 |
| JL125_P_2 | 19 | 1.00 | 20.65 | 19.50 | 0.64 | 1.56 |
| JL125_S_1 | 46 | 1.00 | 48.56 | 51.00 | 0.22 | 4.64 |
| JL125_S_2 | 25 | 1.00 | 56.16 | 39.00 | 0.24 | 4.10 |

| **Supplementary Table S2.** Pure culture sequencing data for strain MM125_6 | | | | | | | | |
| --- | --- | --- | --- | --- | --- | --- | --- | --- |
| Sample | Complete  (%) | Contamination  (%) | Raw data pair reads | Raw data total bases (bp) | Raw data  Q20  (%) | Clean data  pair reads  (bp) | Clean data  total bases  (bp) | Clean data  Q20  (%) |
| MM125-6 | 100 | 0.2323 | 10535178 | 1590811878 | 0.972792 | 10272098 | 1548784954 | 0.979938 |

| **Supplementary Table S3.** Genome features of *Alcanivorax* sp. MM125_6 | |
| --- | --- |
| Genome features | |
| Genome size (bp) | 4,097,225 |
| G+C content (mol%) | 66.13 |
| Contigs | 124 |
| Scaffold N50 (bp) | 184,497 |
| Total number of CDS(Codons) | 3,827 |
| tRNA | 47 |
| rRNA | 8 |
| CRISPR arrays | 7 |
| KEGG metabolic pathways | 2268 |
| Genes involved in xenobiotics biodegradation | 27 |
