# Supplementary figures and images for: Potential autotrophic carbon-fixer and Fe(II)-oxidizer Alcanivorax sp. MM125-6 isolated from Wocan hydrothermal field
Source: Front Microbiol. 2022 Oct 14;13:930601. doi: 10.3389/fmicb.2022.930601 (PMC9616709; doi:10.3389/fmicb.2022.930601)

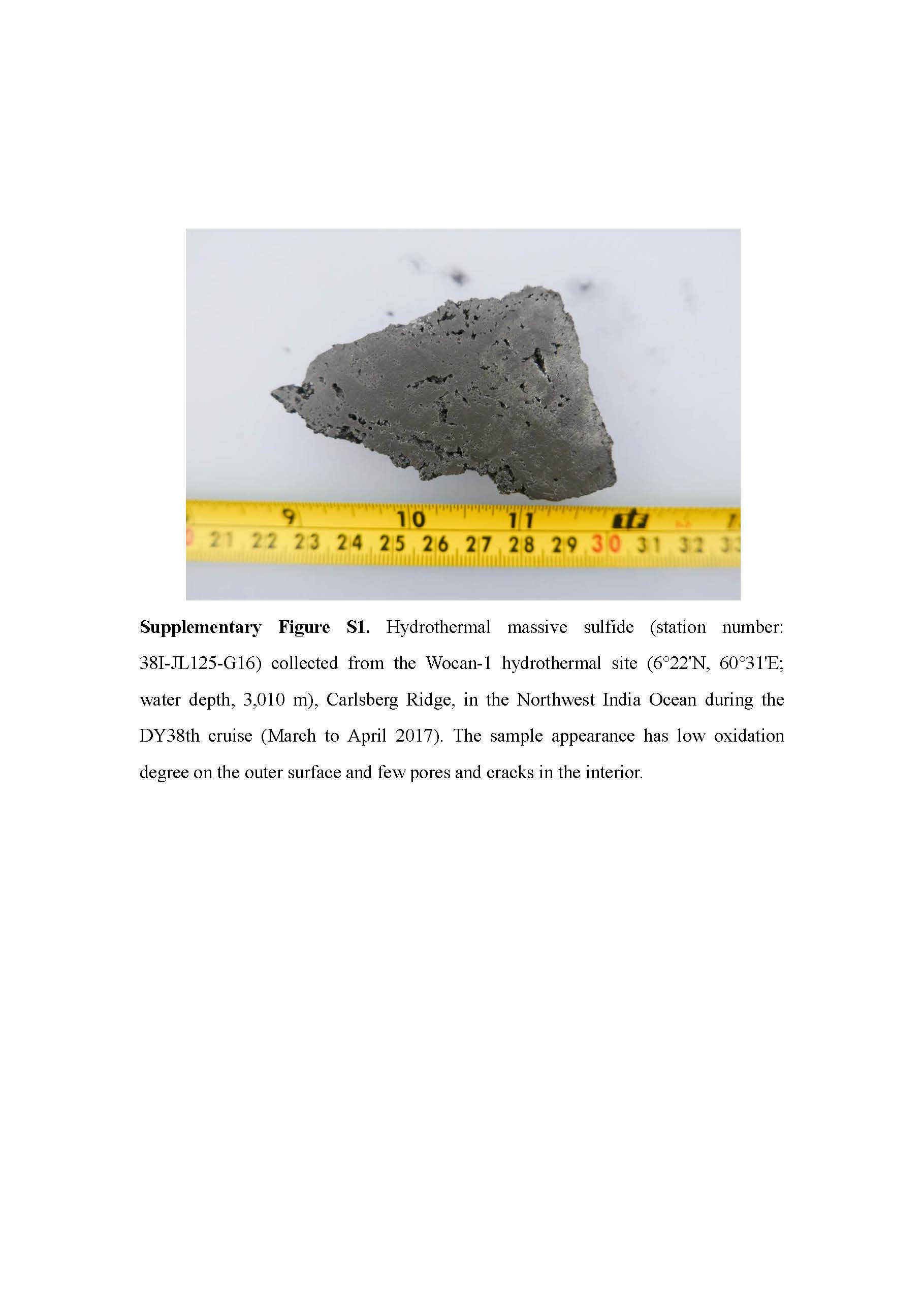

Supplement: Supplementary file 1 [file Data_Sheet_1.zip › Image_1.JPEG]

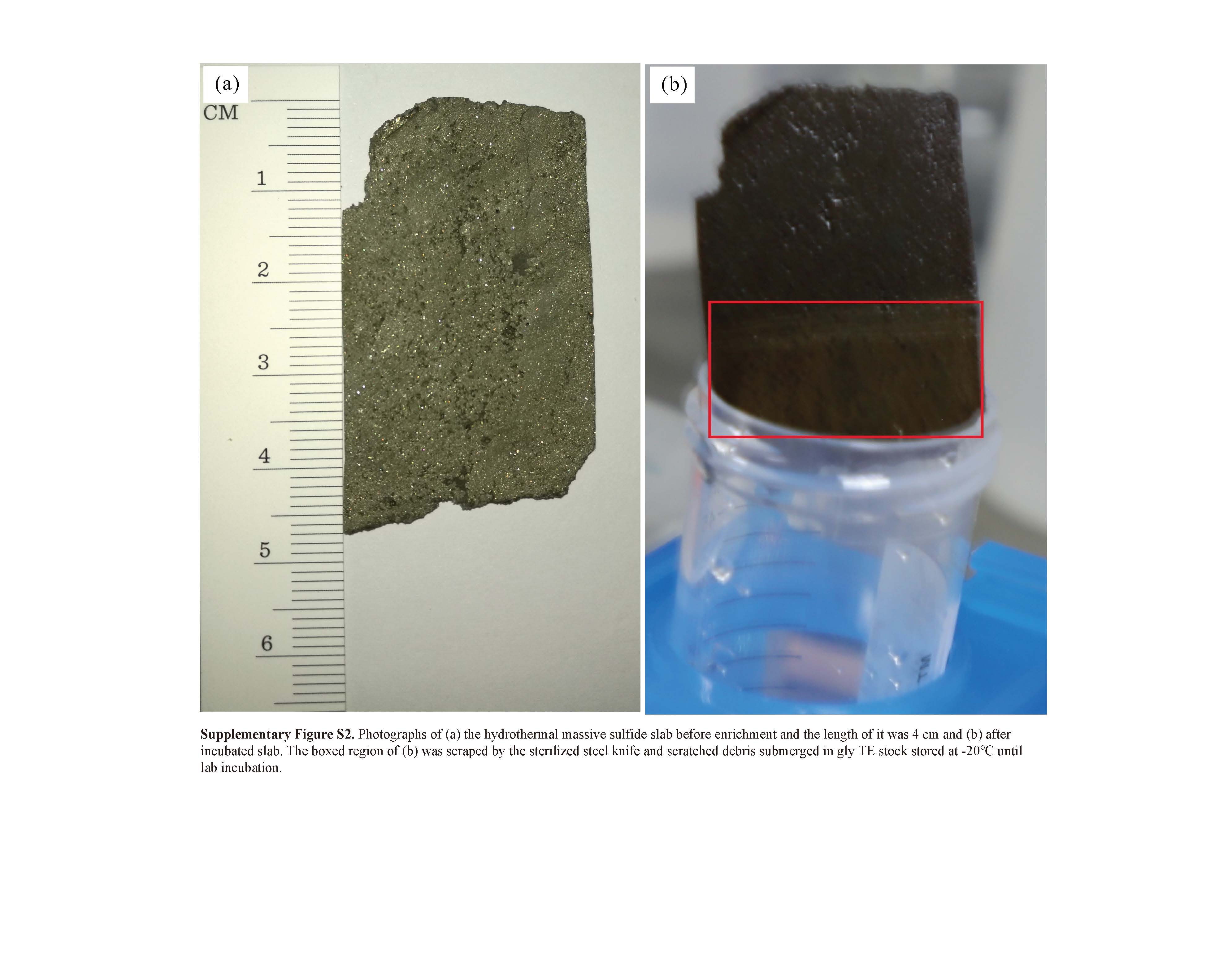

Supplement: Supplementary file 1 [file Data_Sheet_1.zip › Image_2.JPEG]

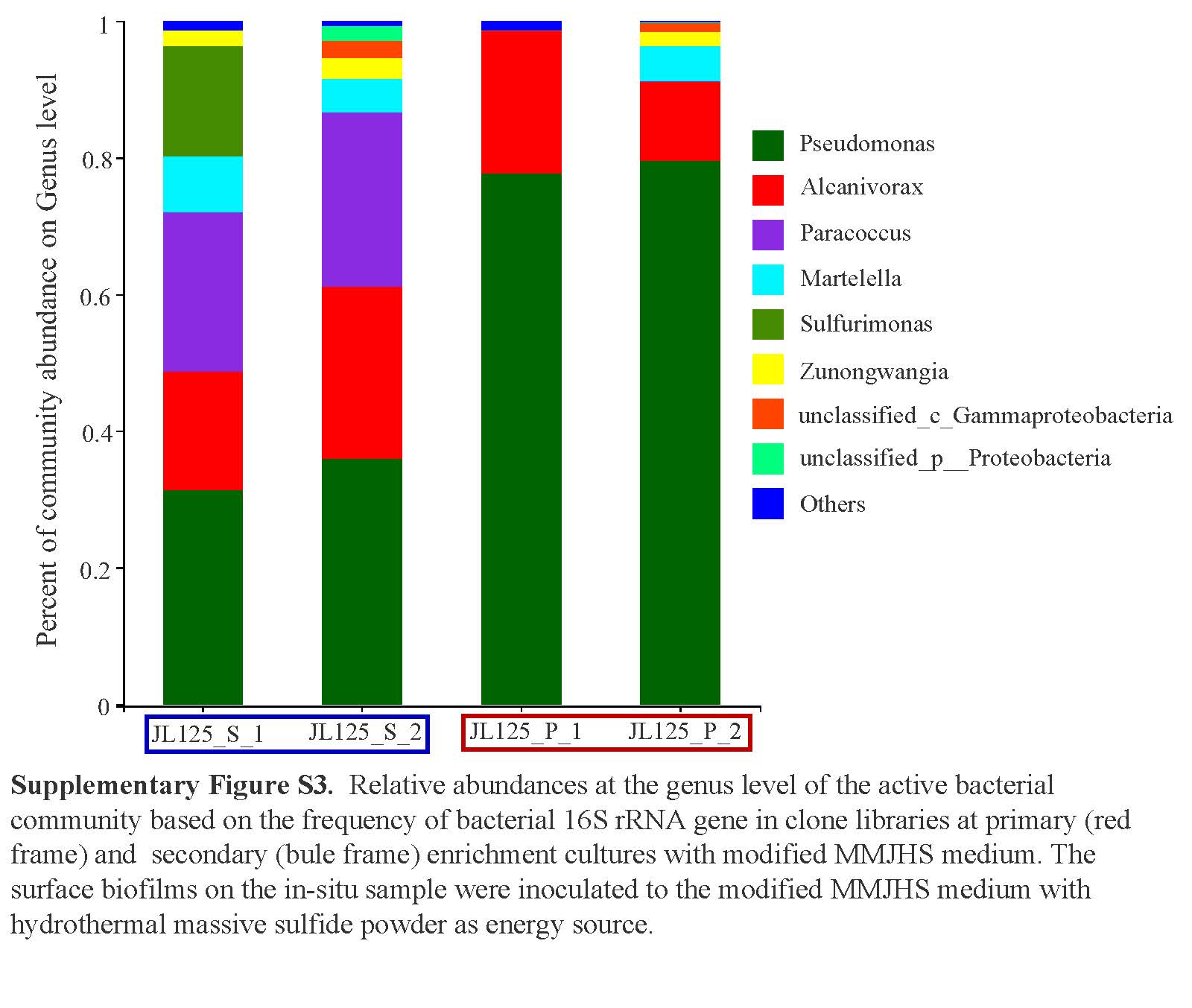

Supplement: Supplementary file 1 [file Data_Sheet_1.zip › Image_3.JPEG]

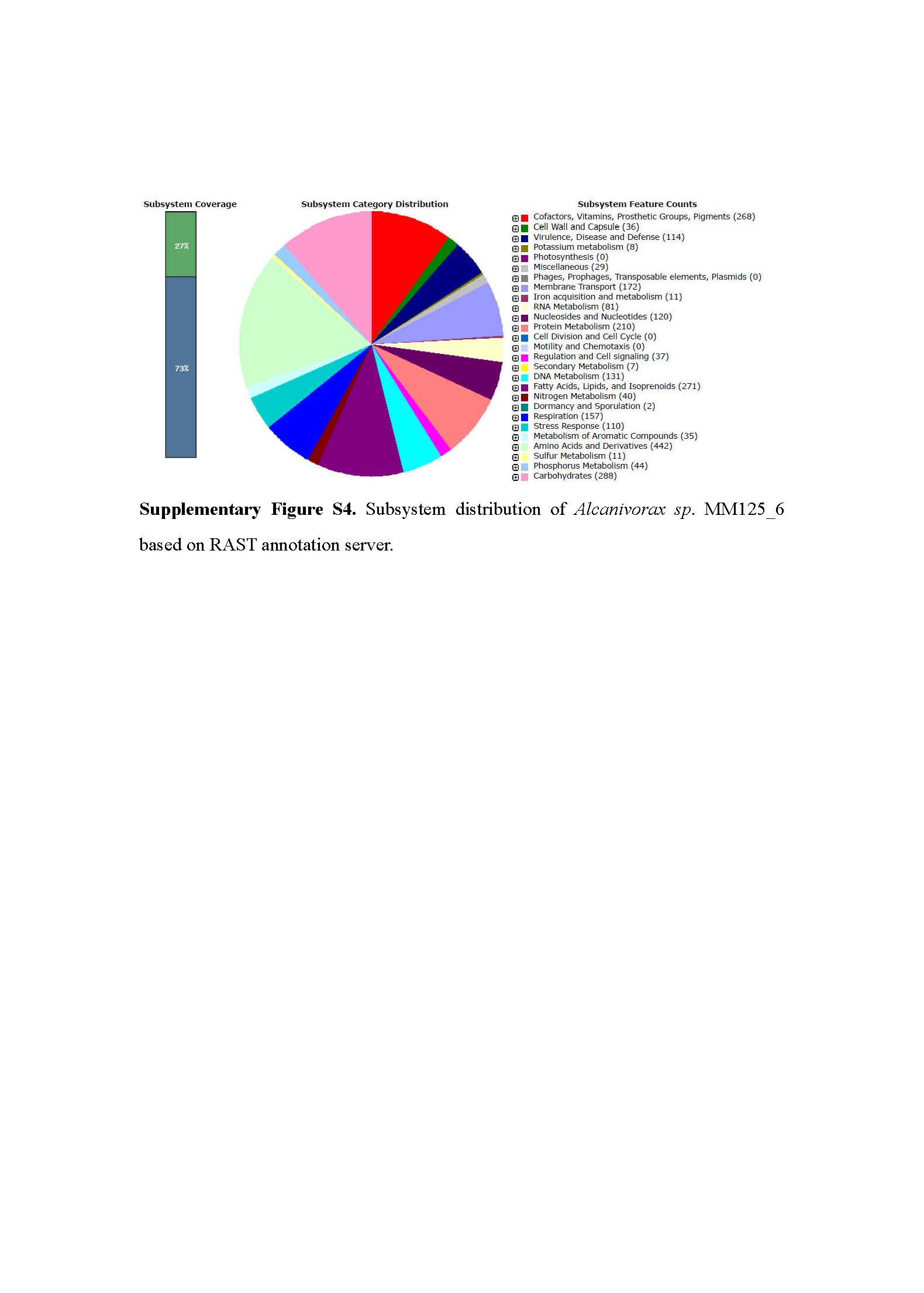

Supplement: Supplementary file 1 [file Data_Sheet_1.zip › Image_4.JPEG]

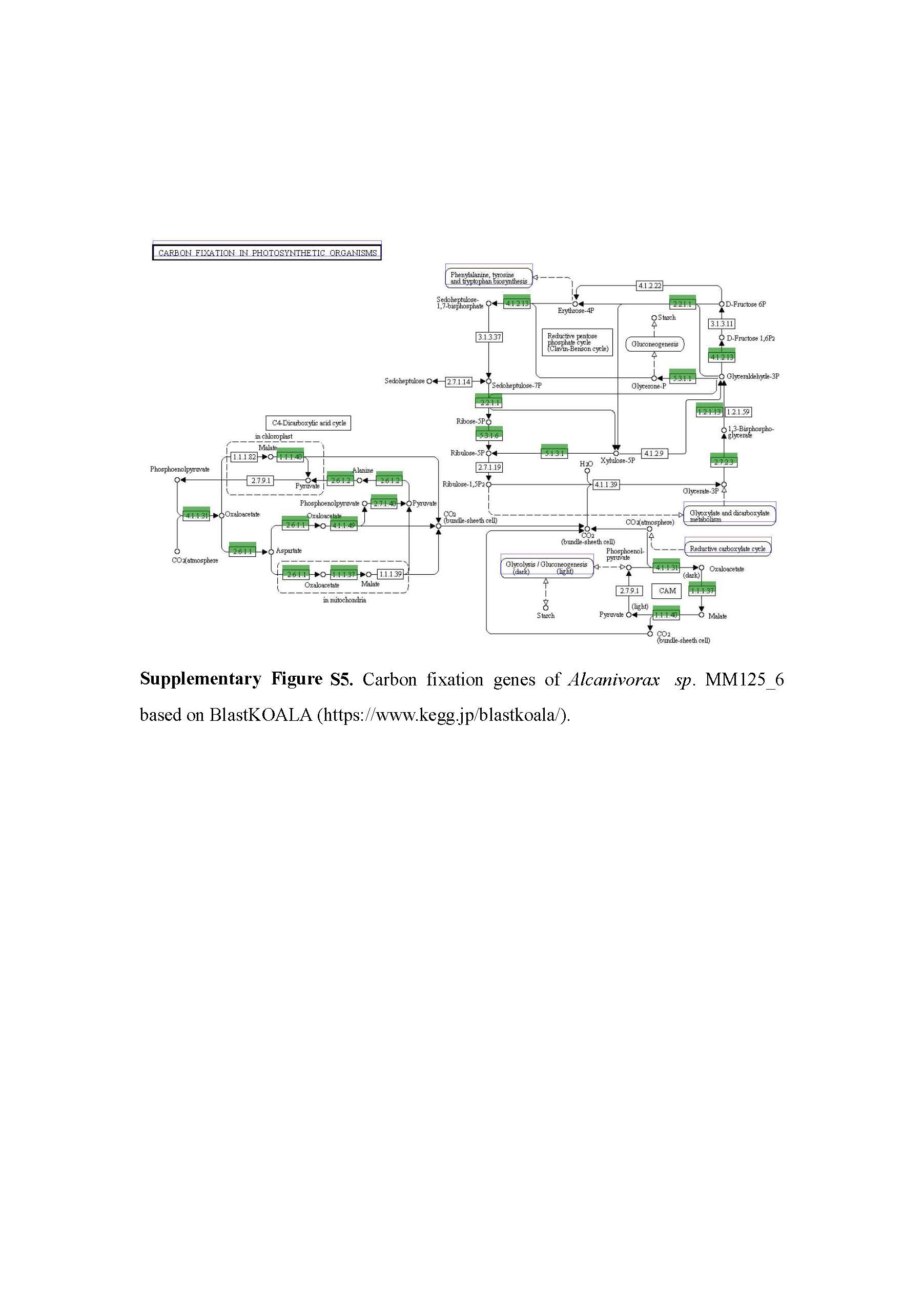

Supplement: Supplementary file 1 [file Data_Sheet_1.zip › Image_5.JPEG]

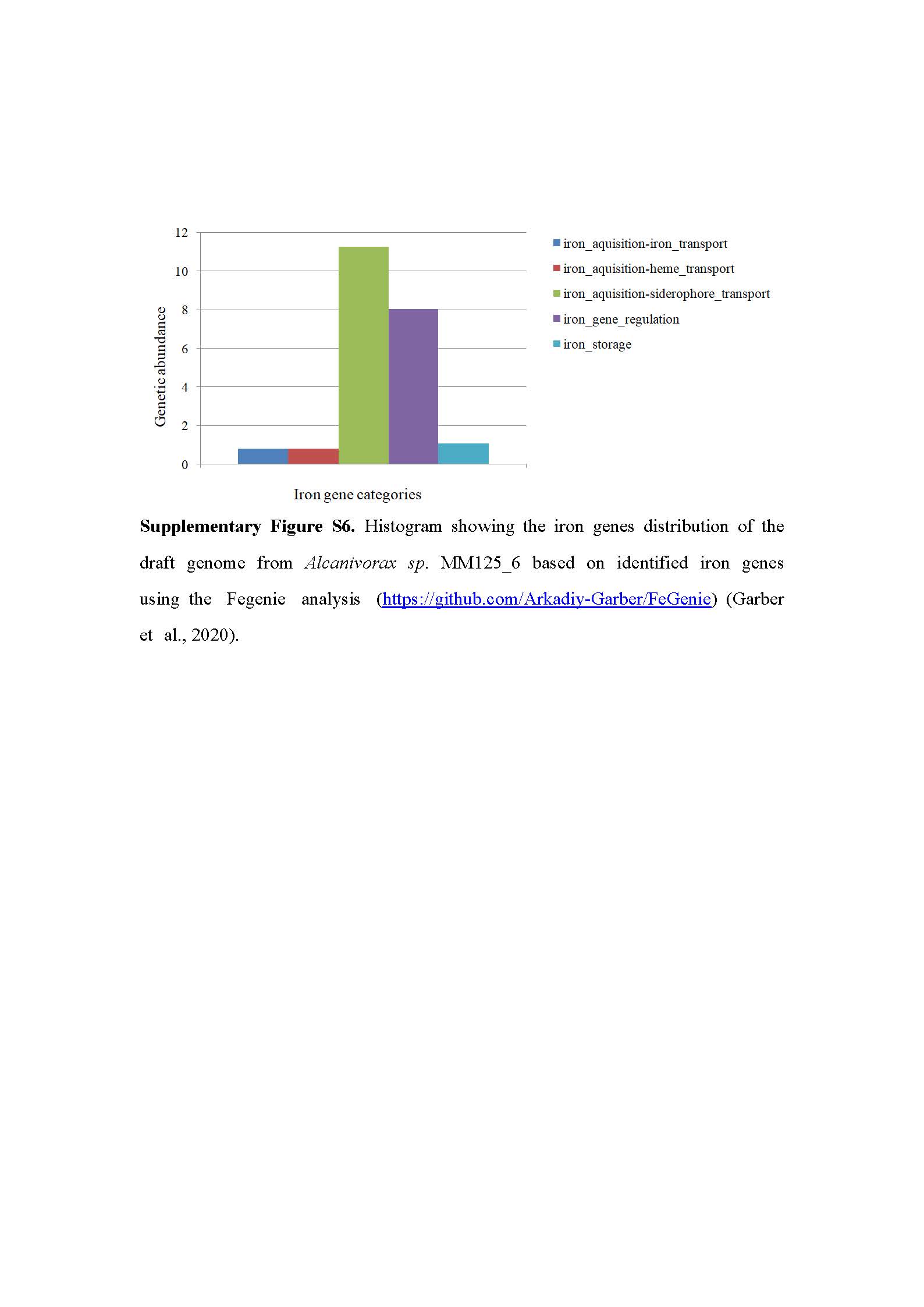

Supplement: Supplementary file 1 [file Data_Sheet_1.zip › Image_6.JPEG]

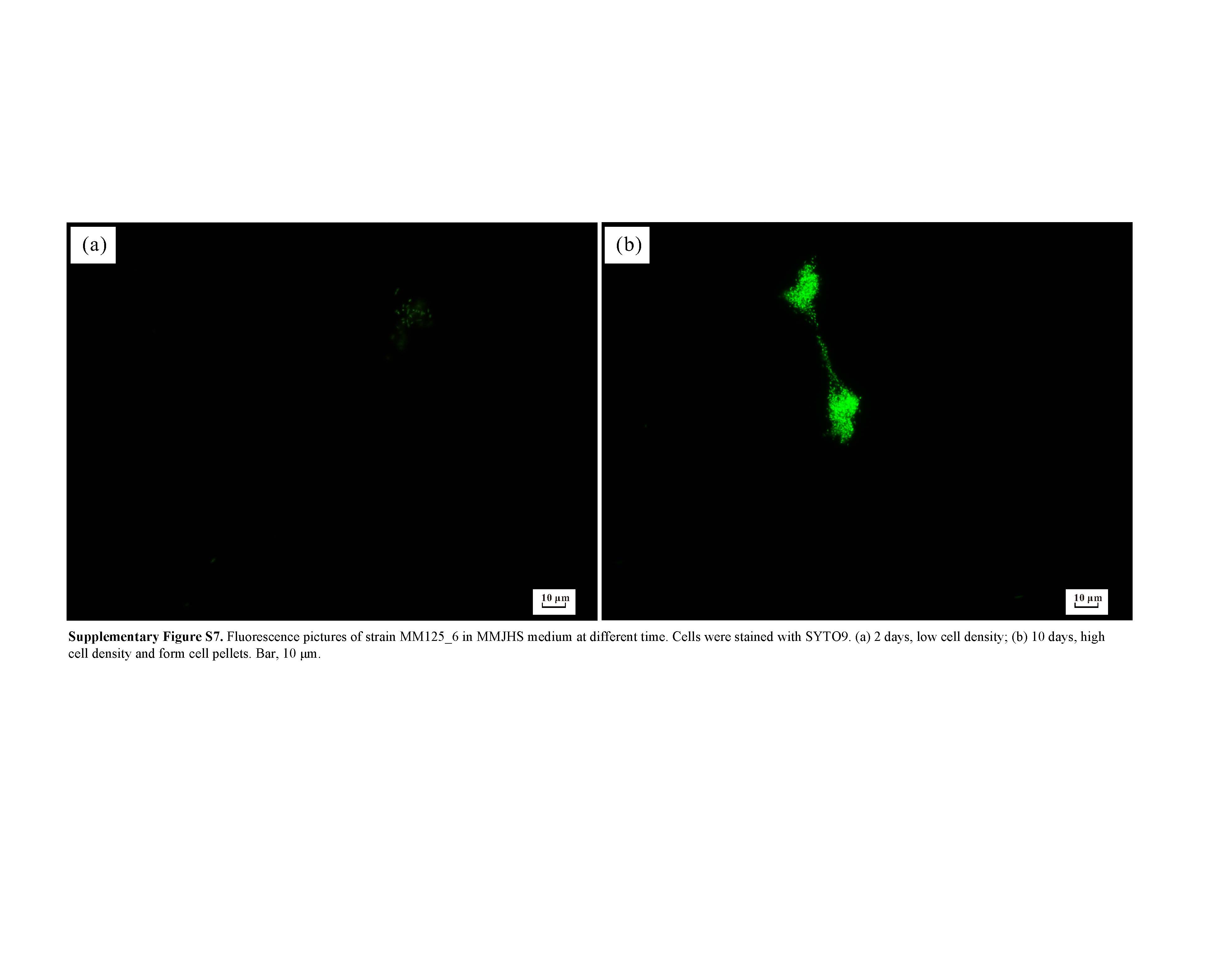

Supplement: Supplementary file 1 [file Data_Sheet_1.zip › Image_7.JPEG]
